# Nuclear Lipid Microdomain as Place of Interaction between Sphingomyelin and DNA during Liver Regeneration

**DOI:** 10.3390/ijms14046529

**Published:** 2013-03-25

**Authors:** Elisabetta Albi, Andrea Lazzarini, Remo Lazzarini, Alessandro Floridi, Eleni Damaskopoulou, Francesco Curcio, Samuela Cataldi

**Affiliations:** 1 Laboratory of Nuclear Lipid BioPathology, Research Center of Biochemical-Specialized Analyses, 06100 Perugia, Italy; E-Mails: Remo30@libero.it (R.L.); direzione@crabion.it (A.F.); hdamaskopoulou@yahoo.gr (E.D.); samuelacataldi@libero.it (S.C.); 2 Department of Clinical and Biological Sciences, University of Udine, 33100 Udine, Italy; E-Mails: andrylazza@gmail.com (A.L.); curcio@uniud.it (F.C.)

**Keywords:** nucleus, nuclear lipid microdomains, sphingomyelin, sphingomyelinase, sphingomyelin-synthase, cell proliferation

## Abstract

Nuclear sphingomyelin is a key molecule for cell proliferation. This molecule is organized with cholesterol and proteins to form specific lipid microdomains bound to the inner nuclear membrane where RNA is synthesized. Here, we have reported the ability of the sphingomyelin present in the nuclear microdomain to bind DNA and regulate its synthesis, and to highlight its role in cell proliferation induced by partial hepatectomy. During G1/S transition of the cell cycle, sphingomyelin and DNA content is very high and it is strongly reduced after exogenous sphingomyelinase treatment. During the S-phase of the cell cycle, the stimulation of sphingomyelinase and inhibition of sphingomyelin–synthase are accompanied by the DNA synthesis start. To assess the specificity of the results, experiments were repeated with trifluoperazine, a drug known to affect the synthesis of lipids and DNA and to stimulate sphingomyelinase activity. The activity of sphingomyelinase is stimulated in the first hour after hepatectomy and sphingomyelin–DNA synthesis is strongly attenuated. It may be hypothesized that the nuclear microdomain represents a specific area of the inner nuclear membrane that acts as an active site of chromatin anchorage thanks to the stabilizing action of sphingomyelin. Thus, sphingomyelin metabolism in nuclear lipid microdomains is suggested to regulate cell proliferation.

## 1. Introduction

The eukaryotic cell cycle has an established process at the end of which one cell forms two genetically identical cells. This means that the genetic material must be duplicated before the division and equally subdivided between the daughter cells. This can be realized through “mechanical” events related to DNA replication, mitosis, cytokinesis and “regulatory” events which control the transit of the cells through the various phases of cell cycle [1]. Liver regeneration (LR) represents a good model of synchronized proliferation *in vivo* that is very useful for studying events correlated with various phases of the cell cycle. The hepatocytes have low mitotic activity in adult rats and acquire the ability to divide during LR following partial hepatectomy (PH) re-entering rapidly in the cell cycle from the G0-phase [2,3]. G0/G1 phase transition occurs 4–6 h after PH, whereas cell proliferation 6–66 h after PH, characterized by G1/S phase transition at 6–12 h, DNA synthesis (S phase) at 18 h, S/G2 phase transition at 18–24 h and first cell division at 24 h after PH [2,3]. For cell differentiation and liver tissue structure, functional rebuilding occurs 72–168 h after PH [2]. The process of LR has been widely studied since the 1960s, demonstrating the importance of different regulatory proteins, growth factors and hormones [4,5]. DNA synthesis and cell cycle during LR have also been extensively studied [6–10] Recently, Xu *et al*. have highlighted the relevance between lipid metabolism-associated genes and LR demonstrating that 280 genes involved in lipid metabolism are proven to be LR-associated [2]. In particular, gene expression of sphingomyelin (SM) metabolism enzymes change 2–16 h after PH [2]. It is consistent with the observation that PH induces production of pro-mitogenic intermediates of SM signaling pathway during the first 12 h of LR in rat [11], particularly due to the activation of neutral-sphingomyelinase (N-SMase) [12], which precedes the maximum of DNA synthesis [13]. Because the first hours after PH are characterized by DNA and RNA synthesis and the enzymes for SM metabolism localized inside the nucleus are enzymatically active, we sought to demonstrate that intranuclear SM cycle worked independently from that of the cell and nuclear membrane during the cell cycle [14]. The decondensation of chromatin in G1/S phase transition is favored by the activation of N-SMase localized near the duplicating DNA. In contrast, synthesis of SM is observed at the end of S-phase for activation of sphingomyelin–synthase (SM-synthase) [14]. Moreover we have recently noticed that part of nuclear SM is structured with cholesterol (CHO) and proteins to form the nuclear lipid microdomain (NLM) associated with the inner nuclear membranes [15]. Our first observation conduced at 24 h after PH showed an increase of SM in NLM in association with an enrichment in labeled uridine incorporation, thus indicating that this structure acts as a platform for the transcription process [15,16]. The present paper was conceived of our strong desire to better study the behavior and the specificity of SM present in NLM during the phases of the cell cycle. To this end, LR has been used as an experimental model and analyses were conducted on NLM purified at different times after surgery. To analyze the relationship between SM and DNA, the trifluoperazine, a drug known to affect the synthesis of lipids and DNA [17], was used. Trifluoperazine is a potent, competitive inhibitor of the calcium–calmodulin complex that, when given to rats after partial hepatectomy, causes a significant decrease in DNA synthesis [18]. Its injection at 4 h after PH produces a strong inhibition of DNA synthesis observed at 24 h after surgery; but when injection is administered at 20 h after PH, it does not produce an effect on DNA replication [19]. The trifluoperazine inhibits the incorporation of [^14^C] acetate into lipids [17] and stimulates SMase activity [20]. In the present work, we provide evidence for the existence of NLM as a place for SM–DNA interaction during G1/S transition of cell cycle. The trifluoperazine slows the synthesis of SM and stimulates the SMase activity during G1-phase and inhibits DNA synthesis during the S-phase of cell cycle.

## 2. Results and Discussion

### 2.1. Nuclear Lipid Microdomains during Liver Regeneration

**Figure 1:**
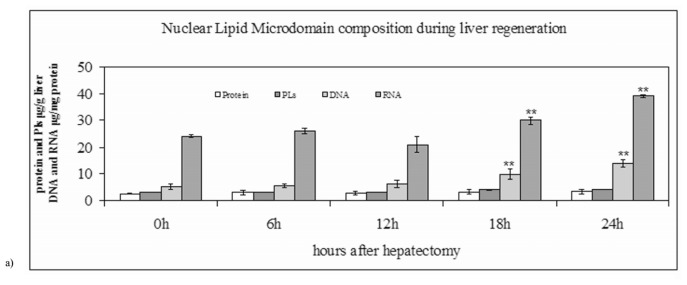

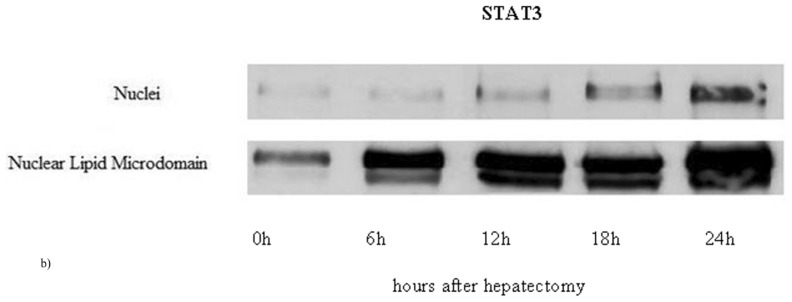
(**a**) Protein, phospholipids (PLs), DNA and RNA content in nuclear lipid microdomain during liver regeneration. The values were expressed as μg/mg liver for protein and PLs and μg/mg protein for DNA and RNA and represented the average ± SD of three experiments performed in duplicate. (* *p* < 0.001 *versus* 0 h); (**b**) STAT3 in nuclei and nuclear lipid microdomain during liver regeneration. The amount corresponding to 30 μg proteins was loaded onto SDS-PAGE electrophoresis in 12% polyacrylamide slab gel. Immunoblot of proteins were probed with anti-STAT3 (apparent molecular weight 90 KDa) antibody and visualized by ECL. At the bottom, one will find the number of hours after partial hepatectomy.

**Figure 2:**
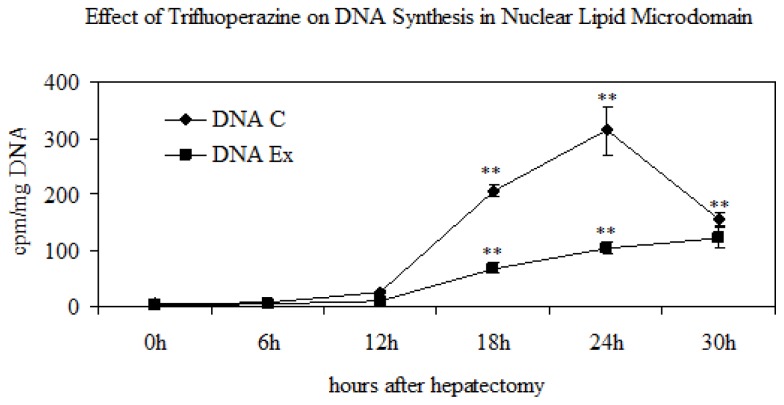
DNA synthesis in nuclear lipid microdomain during rat liver regeneration: effect of trifuoperazine. The rat liver was stimulated to proliferate by partial hepatectomy corresponding to 75% of rat liver. The rats were killed at regular intervals between 0 and 30 h after hepatectomy. The specific activity was calculated as cpm/μg DNA. The data represent the media ± SD of three separated experiments performed in duplicate. Significance * *p* < 0.001 *versus* 0 h.

At 0 h after surgery, the NLM composition was similar to that of normal rat liver previously described [15]. The protein and PL content was 2.71 ± 0.04 and 3.21 ± 0.07 μg/mg liver, respectively and the ratio among CHO, PC and SM was approximately to 1:1:1. The content of nucleic acids was very low. In fact, the content of RNA was 23.96 μg/mg protein and that of DNA was 5.16 μg/mg protein. The analysis of STAT3 as a specific marker of NLM [15] showed the high level of purification (see 0 h, Figure 1b). To study the characteristics of NLM during LR, the animals were killed at regular intervals after operation from 0 to 24 h. The incorporation of labeled thymidine into DNA was used to test cell cycle (Figure 2), as previously reported [6–10]. The protein content did not change significantly during LR (Figure 1a), whereas the PL, DNA and RNA content increased 1.3, 2.7 and 1.6-folds, respectively, at 24 h after PH (Figure 1a). The STAT3 protein increased progressively, particularly at 12 h, 18 h and 24 h during the S-phase of the cell cycle (Figure 1b), as previously reported [21].

### 2.2. Sphingomyelin in Nuclear Lipid Microdomain during Liver Regeneration

It has been reported that the nuclear SM is localized in NM, nuclear matrix, chromatin, and nucleolus [22] and that it has different roles in relation to its localization. In fact, in NM and in the nuclear matrix, the SM was responsible for the maintenance of normal fluidity in no stimulated cells [23]. During rat LR, the modification of SM content changed the fluidity of NM, thus favoring an increase of mRNA transport and of nuclear matrix favoring the relaxation of the superhelical strain, as well as the processing of hnRNA and snRNP, and RNA transport [24] and regulated the DNA synthesis during liver regeneration [23].

Since the aim of the paper was to understand the role of SM present in NLM as a specific section of nuclear SM, the behavior of this molecule was studied during LR and the results compared with those of SM localized in NM, nuclear matrix, and chromatin. Table 1 confirmed that the nuclear SM is strongly concentrated in NLM, as previously reported [15], and showed that its increase, at 12 h after PH when G1/S transition of the cell cycle starts, was similar to that observed in chromatin. No variations were reported in sham-operated animals. Our data showed for the first time that in the NLM not only occurs in RNA synthesis, as previously reported [15], but also DNA synthesis (Figure 2), suggesting that this structure represents an attachment site for active chromatin, and its plasticity influences nuclear function. On the other hand, ultrastructural cytochemical study on the intranuclear distribution of SM has indicated that this molecule is associated with transcriptionally active chromatin and the microinjection of enzymatically active SMase into living cells resulted in a rapid degradation of intranuclear structure [25]. Previous observations have shown the existence of two intranuclear pools of SM: one CHO-free and another CHO-linked fraction [3], and the last fraction is present in NLM [15].

**Figure 3:**
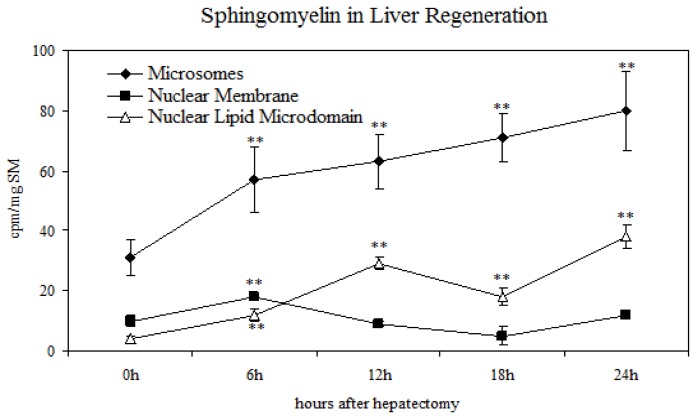
Incorporation of 32P in sphingomyelin of microsome, nuclear membrane and nuclear lipid microdomain during liver regeneration. The data are expressed as cpm/mg SM and represent the average ± SD of four experiments performed in duplicate (* *p* < 0.001 *versus* 0 h).

To analyze the plasticity of NLM in relation to cell proliferation, SM synthesis and breakdown were studied by injection of the rats with 100 μC [^32^P]O_4_^2−^ 1 h prior to killing. The incorporation of labeled precursor in SM was evaluated in the purified M, NM and NLM. The results showed that in M and NM, SM metabolism was not correlated with the cell cycle (Figure 3). In fact, in both structures, the value increased at 6 h after PH and, then, while in M, remained practically constant; in NM, it decreased slowly, eventually reaching the value of 0 h during the following hours. In contrast, the SM of NLM showed a peak at 12 h, when the G1/S transition of the cell cycle starts, followed by a decrease at 18 h and a new increase at 24 h. The variations observed in sham-operated animals were not significant.

**Figure 4:**
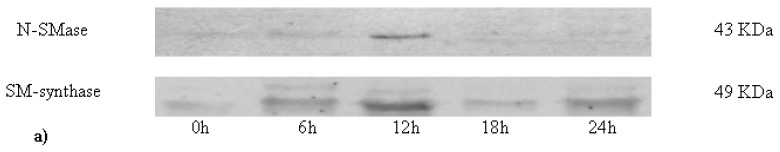

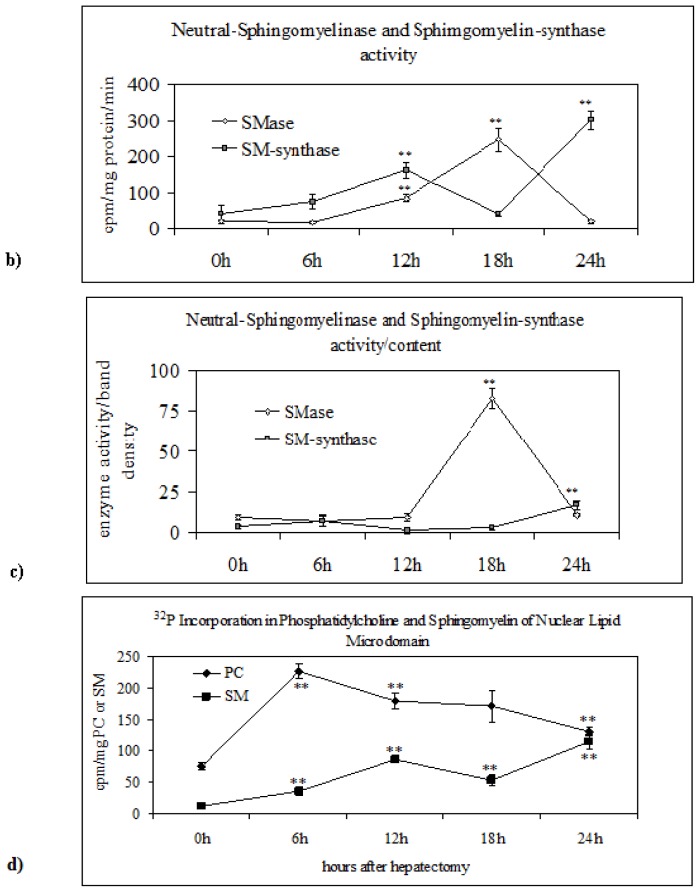
Sphingomyelin metabolism in nuclear lipid microdomain during liver regeneration. (**a**) SMase and SM-synthase content; the amount corresponding to 30 μg proteins were loaded onto SDS-PAGE electrophoresis in 8% polyacrylamide slab gel. Immunoblot of proteins were probed with anti-SMase (apparent molecular weight 43 KDa) or with anti-SM-synthase (apparent molecular weight 49 KDa) antibodies and visualized by ECL; (**b**) SMase and SM-synthase activity; the enzyme activity is expressed as cpm/mg protein/min; (**c**) SMase and SM-synthase activity referred to the value of band density evaluated as reported in “Materials and Methods.” The data represent the average ± SD of four experiments performed in duplicate (* *p* < 0.001 *versus* 0 h); (**d**) Incorporation of ^32^P in sphingomyelin and phosphatidylcholine present in nuclear lipid microdomain. The data represent the average ± SD of four experiments performed in duplicate (* *p* < 0.001 *versus* 0 h).

To study whether the variation of the SM of NLM in relation to the cell cycle was due to the modification of the enzyme for SM metabolism, the N-SMase and SM-synthase were studied. The Figure 4a showed that, after PH, the band corresponding to 43 KDa’s apparent molecular weight, highlighted by anti-SMase antibody, was strongly evident only at 12 h after surgery and it was reduced immediately until 24 h. The enzyme activity, measured as cpm/mg protein/min, showed a peak at 18 h after PH (Figure 4b). To exclude that this high value could be due to the high value of the enzyme content, the value of enzyme activity was referred to the band density of the immunoblotting analysis. In this way, the strong peak at 18 h of the N-SMase activity was confirmed (Figure 4c). Alternatively, the immunoblotting band corresponding to 49 KDa’s apparent molecular weight, highlighted by anti-SM-synthase, was more colored at 6 h and 24 h in comparison to 0 h, and it was strongly evident at 12 h after PH (Figure 4a). The SM-synthase enzyme activity showed two peaks at 12 and 24 h (Figure 4b). However, whether or not the activity referred to band density, its value increased only at 24 h (Figure 4c). All modifications described were absent in sham-operated animals. Since N-SMase degrades SM and SM-synthase synthesizes SM by using the phosphocholine of PC, to confirm the results of enzyme activity, the incorporation of [^32^P]O_4_^2−^ in these lipids was analyzed. The peak of N-SMase activity could justify the reduction of SM labeled at 18 h, whereas the increase of SM-synthase activity at 24 h could justify the reduction of labeled PC and the increase of labeled SM at this time (Figure 4d). All variations of enzyme activities and ^32^P incorporation in lipids in relation to cell cycle were absent in sham-operated animals.

### 2.3. Effect of Trifluoroperazine Treatment

**Figure 5:**
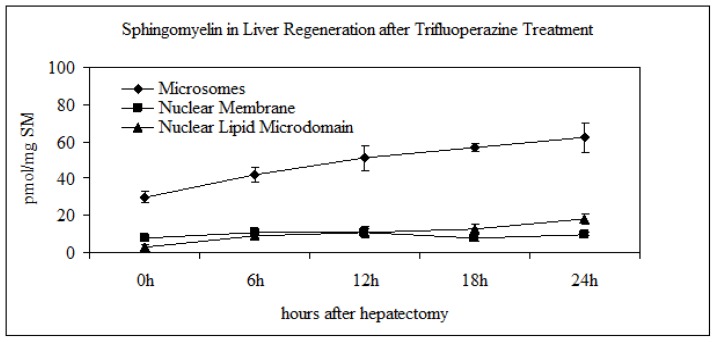
Effect of trifluoperazine on the incorporation of ^32^P in sphingomyelin of microsome, nuclear membrane and nuclear lipid microdomain during liver regeneration. The data are expressed as cpm/mg SM and represent the average ± SD of four experiments performed in duplicate.

**Figure 6:**
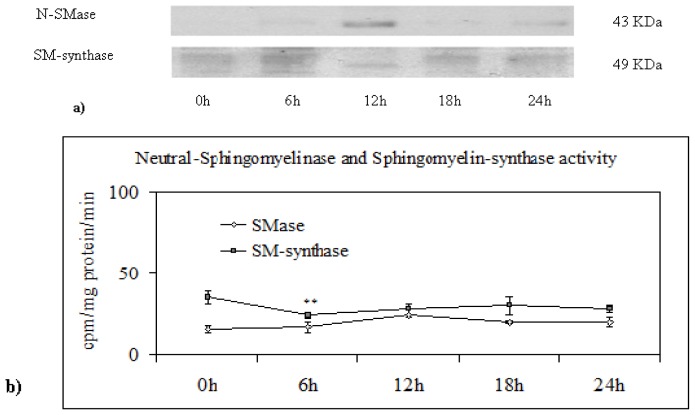

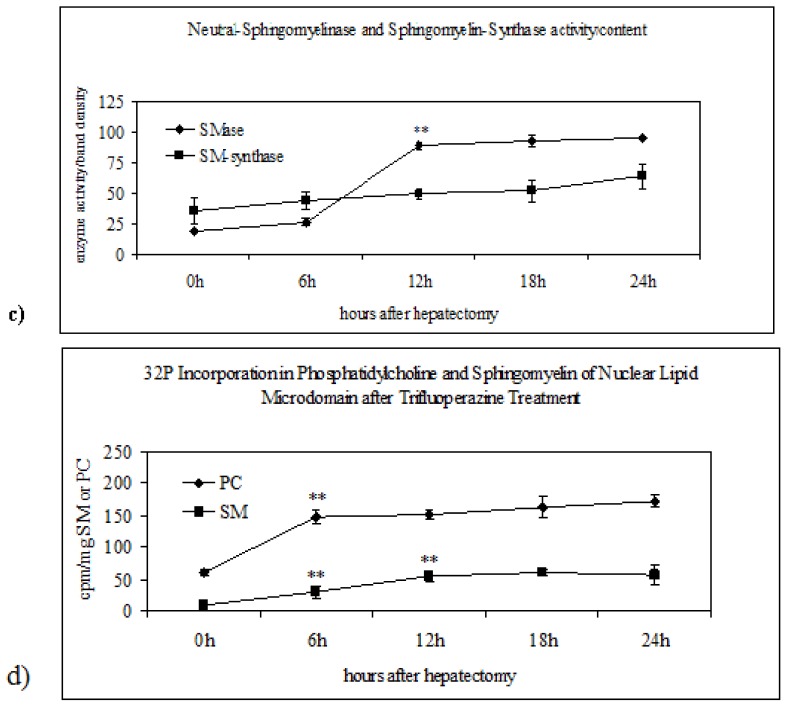
Effect of trifluoperazine on sphingomielin metabolism in nuclear lipid microdomain during liver regeneration. (**a**) SMase and SM-synthase content. (see legend Figure 3); (**b**) SMase and SM-synthase activity; the enzyme activity is expressed as cpm/mg protein/min; (**c**) SMase and SM-synthase activity referred to the value of band density (see legend Figure 3). The data represent the average ± S. of four experiments performed in duplicate. (* *p* < 0.001 *versus* 0 h); (**d**) Incorporation of ^32^P in sphingomyelin and phosphatidylcholine present in nuclear lipid microdomain. The data represent the average ± SD of four experiments performed in duplicate (* *p* < 0.001 *versus* 0 h).

To confirm the relation of the SM metabolism in NLM and the cell cycle, the experiments were repeated after intraperitoneal injection of the hepatectomized or sham-operated rats with trifluoperazine that inhibits the DNA synthesis. The kinetics of the DNA synthesis was studied by ^3^H-thymidine incorporation. The specific activity of the DNA, calculated as cpm/μg DNA, was very low at 6 and 12 h after PH; it increased strongly at 18 and 24 h (Figure 2), thus corresponding to the S phase of the cell cycle and supporting previously results obtained in isolated chromatin [22]. The treatment with trifluoperazine induced 67% reduction of the DNA synthesis (Figure 2). This drug determined low variations of ^32^P incorporation in SM present in M and NM, whereas the variation of the incorporation in SM of NLM in relation to the cell cycle was cancelled (Figure 5). No changes were present in sham-operated animals. This result was due to the different content of SM-synthase (Figure 6a), to the inhibition of the peaks of the N-SMase and SM-synthase activities either evaluated alone (Figure 6b) or related to the band density (Figure 6c), thus inhibiting PC and SM change in relation to cell cycle (Figure 6c). Our data demonstrate the role of SMase and SM-synthase activity in the metabolism of SM present in NLM by changing its plasticity and consequently its function during cell proliferation.

## 3. Experimental Section

### 3.1. Materials

Radioactive [Me-^14^C] SM (54.5 Ci/mol, 2.04 GB q/mmol), [Me-^3^H] (L-3-phosphatidyl [N-Me-^3^H] choline 1,2 dipalmitoyl, 81.0 Ci/mmol, 3.03 TB q/mmol) and [^3^H] thymidine (41 Ci/mmol, 1.52 TB q/mmol) were from Amersham Pharmacia Biotech (Rainham, Essex, UK), Ecoscint A from National Diagnostic (Atlanta, GA, USA). Chemicals, bovine serum albumin, PC, SM, ceramides, trifluoperazine, phenylmethylsulfonylfluoride (PMSF), ethylendiaminetetracetic acid (EDTA), IgG peroxidase coniugate were from Sigma Chemical Co. (St. Louis, MO, USA). TLC plates (silica gel G60) were from Merck, Darmstadt, Germany. Anti-signal transducer and activator of transcription-3 (STAT3), anti N-SMase and anti-SM-synthase 1 were obtained from Santa Cruz Biotechnology, Inc. (Dallas, TX, USA). SDS-PAGE Molecular Weight Standard (Bio-Rad Laboratories, Hercules, CA, USA).

### 3.2. Animals

Sixty-day-old Sprague Dawley female rats (Harlan Nossan, Milan, Italy), kept at normal light–dark periods, were used. They had free access to pelleted food and water.

### 3.3. Liver Regeneration

To study the role of SM metabolism in NLM during cell proliferation, LR was induced by PH corresponding to 75% of total liver, which stimulates liver cells to proliferate. Sham-operated animals were used as control. The animals were laparatomized after anesthesia with 100 mg/Kg Farmotal (Farmitalia, Italy) and the hepatectomy was performed between 8 and 10 a.m. The animals were killed at regular intervals after operation from 0 to 24 h by cervical dislocation under anesthesia. To analyze the lipid synthesis and breakdown, rats were injected with 100 μC [^32^P]O_4_^2−^ 1 h prior to killing. The incorporation of the labeled precursor was evaluated in SM present in the microsomes (M), nuclear membrane (NM) and NLM. To evaluate the specificity of the results obtained in NLM, the experiments were repeated after intraperitoneal injection of the hepatectomized or sham-operated rats with trifluoperazine. The DNA synthesis was studied by evaluating the incorporation of labeled tymidine in the DNA of NLM from 0 to 30 h. To this end, rats were injected with 100 μC^3^H-tymidine 1 h prior to killing. All treatments were made according to the international regulation of National Institutes of Health.

### 3.4. Microsomes and Subnuclear Fractions Purification

The regenerating liver was homogenized in a Thomas homogenizer with 10 mM Tris-HCl buffer, pH 7.4, containing 0.25 M sucrose, 1 mM EDTA, 0.1% ethanol, 0.2 mM phenylmethylsulfonylfluoride (PMSF) and 0.2 mM dithiothreitol (DTT); the homogenate was filtered through two layers of surgical gauze. The microsomes were isolated by centrifugation at 100.000*g* × 20 min, as previously reported [26]. The nuclear membranes were prepared according to Kay and Johnston [27]. The triton X100-insoluble NLM were purified on sucrose gradient, as previously reported [15].

### 3.5. DNA Synthesis

The DNA synthesis was studied by evaluating the incorporation of labeled tymidine in the DNA present in NLM from 0 to 24 h. The DNA was extracted from NLM and used in part for DNA amount determination [4] and in part for radioactivity evaluation by diluting the samples with 10 mL of Ecoscint A and 1 mL of H_2_O and measuring it with a Packard liquid scintillation counter. The data of the radioactivity referred to the DNA amount and expressed as cpm/μg DNA.

### 3.6. Lipid Analysis

After lipid extraction performed according to Folch *et al*. [28], the upper phase was tried three times with chloroform/methanol (2:1 *v*/*v*) and with solvent containing 0.5% NaCl [3]. The SM and PC were separated on thin-layer chromatography (TLC) by using chloroform/methanol/ammonia (65:25:4 *v*/*v*/*v*) as the solvent system and were localized with iodine vapor on the basis of standards migration. The spots were scraped and used to measure the inorganic phosphorus [29]. The CHO and CHO esters were separated on TLC according to Christie [30], using hexane/diethyl ether/formic acid (80:20:2 *v*/*v*/*v*) as solvent. To study the SM synthesis, [^32^P]O_4_^2−^ incorporation was evaluated in SM present in the microsomes, nuclear membrane and NLM. The SM spot was separated by TLC, diluted with 10 mL Ecoscint A and 1 mL water and the radioactivity was measured with a Packard liquid scintillation analyzer. The data of the radioactivity were referred to the SM amount and expressed as cpm/μg SM.

### 3.7. Sphingomyelinase Treatment

NLM were purified at 12 h after PH. Samples were incubated with 0.1 M Tris-HCl pH 8.4, 6 mM MgCl_2_, 0.1% Triton X-100 in the presence of 5 μL (6 U/mg prot) SMase at 37 °C to a final volume of 0.5 mL. After 60 min, the sample was centrifuged at 15,000 *g* × 20 min. The pellet was recovered and the SM and DNA content was analyzed as reported earlier.

### 3.8. Neutral-Sphingomyelinase and Sphingomyelin-Synthase Activity

N-SMase assay was performed as previously reported [31]. In the reaction, 1 nmol of ^14^C-SM was diluted by adding 49 nmol cold SM to a final activity 1.08 Ci/mol. The reaction mixture contained 0.1 M Tris-HCl pH 8.4, 0.1 mM ^14^C-SM, 6 mM MgCl_2_, 0.1% Triton X-100 and 100 μg protein to a final volume of 0.1 mL. Incubation was performed at 37 °C for 90 min. The reaction was stopped by adding 2 mL chloroform/methanol (2:1 *v*/*v*); 0.4 mL of 0.5% NaCl was added to the tubes under agitation. The next day, the upper phase was removed and diluted in counting vials with 10 mL Ecoscint A and 1 mL water and the radioactivity was measured as already reported.

SM-synthase assay was evaluated as previously reported [31]. Reaction mixtures contained 0.1 M Tris/HCl pH 8.4, 0.25 mM ^3^H-PC (final specific radioactivity 1.27 Ci/mol), 2 mM CaCl_2_, 0.15 mM ceramide, 0.1% Triton X-100 and 100 μg protein, to a final volume of 0.1 mL. Incubation was performed at 37 °C for 45 min. The reaction was stopped by adding 2 mL chloroform/methanol (2:1 *v*/*v*); 0.4 mL of 0.5% NaCl was added to the tubes under agitation. The tubes were centrifuged at 2000 *g* for 10 min and the upper phase was removed. The lower phase was dried under nitrogen flow and the lipids were re-suspended with chloroform. SM was separated with TLC as above reported by adding cold SM to the tubes before chromatography in order to better identify the spot. The SM was localized with iodine vapor and scraped into counting vials with 10 mL Ecoscint A and 1 mL water and the radioactivity was measured as above reported.

### 3.9. Electrophoresis and Western Blot Analysis

About 30 μg of protein from NLM were submitted to SDS-PAGE electrophoresis in 12% and 8% polyacrylamide slab gel for STAT3 and SMase-SMsynthase, respectively. The transfer of protein was carried out into nitrocellulose in 75 min, the membranes were blocked for 30 min with 0.5% no-fatdry milk in PBS, pH 7.5, and incubated over night at 4 °C with specific antibodies. The blots were treated with horseradish-conjugated secondary antibodies for 90 min. Visualization was performed with the enhanced chemiluminescence kit from Amersham. (Rainham, Essex, UK). The apparent molecular weight of the proteins was calculated according to the migration of molecular size standard. The area density of the bands was evaluated by densitometry scanning and analyzed with Scion Image.

## 4. Conclusions

NLM represents an attachment site for active chromatin and its plasticity influences nuclear function. In cell proliferation, the decrease of SM could be responsible for the destabilization of the double-strand DNA, favoring its despiralization and new synthesis. Alternatively, the increase of SM could be responsible for the stabilization of the newly synthetized DNA. Our data suggests that NLM has a specific role in nuclear function during cell life.

## Figures and Tables

**Table 1:** Distribution of sphingomyelin in subnuclear fractions during liver regeneration. The data are expressed as μg/mg protein and represent the mean ± SD. of three independent experiments performed in duplicate.

Time	Nuclear membrane	Nuclear matrix	Chromatin	Nuclear microdomain
0 h	9.3 ± 0.7	1.6 ± 0.3	0.9 ± 0.1	15.5 ± 0.3
6 h	12.8 ± 0.5 *	3.7 ± 0.6 *	0.7 ± 0.2	14.1 ± 0.2
12 h	12.1 ± 1.3 *	6.5 ± 0.5 *	2.2 ± 0.1 *	38.2 ± 0.7 *
18 h	21.3 ± 0.7 *	7.7 ± 0.2 *	0.9 ± 0.1	15.7 ± 0.1
24 h	9.3 ± 0.3	6.8 ± 0.5 *	1.2 ± 0.2	19.5 ± 0.2 *

* *p* < 0.001 *versus* 0 h.
